# Removal efficiencies and environmental risk assessment of selected pharmaceuticals and metabolites at a wastewater treatment plant in Pietermaritzburg, South Africa

**DOI:** 10.1007/s10661-024-13515-z

**Published:** 2024-12-26

**Authors:** Nikitha Inarmal, Brenda Moodley

**Affiliations:** School of Chemistry and Physics, University of KwaZulu-Natal, Westville Campus, Durban, 4000 South Africa

**Keywords:** Wastewater treatment plant, Removal efficiency, Environmental risk assessment, Ivermectin, Pharmaceuticals, Metabolites

## Abstract

This research study critically evaluates the concentrations of selected pharmaceuticals found within wastewater and at various stages within a selected wastewater treatment plant. The study further investigates the effects of seasonal variation, between wet and dry months, on the removal of target analytes. To the best of the authors’ knowledge, ivermectin in wastewater has not been investigated in South Africa. The methodology used was extraction of the wastewater samples using solid phase extraction with Oasis HLB cartridges followed by analysis on a liquid chromatography-mass spectrometer. As expected, analyte concentrations were observed to decrease as wastewater went through the treatment process with the greatest reduction in concentrations observed between secondary effluent and maturation samples, which could be indicative of photodegradation of compounds. Influent concentrations of analytes ranged from 0.008184 to 1.243 mg L^−1^ while maturation river concentrations ranged from not detected to 0.9286 mg L^−1^. Removal efficiencies ranged from 7.70 to > 99.99% of analyte removed. Significant differences in removals were observed between the wet and dry seasons experienced within the province. Environmental risk assessment ratios ranged from 0.0863 to 10.5 × 10^8^. Ratios for metformin and valsartan were well below 1, indicating that they would not be of environmental risk. While ratios for ivermectin were substantially high, ranging from 25.4 × 10^7^ to 10.5 × 10^8^ indicating that it is of severe environmental risk and is of significant concern. Generally, the analyte concentrations quantified within influent samples were higher than effluent samples which is indicative of the treatment process being able to partially remove some of the analytes. Risk assessment ratios suggest high risk of selected pharmaceuticals which are of environmental concern.

**Supplementary Information:**

The online version contains supplementary material available at 10.1007/s10661-024-13515-z.

## Introduction

Emerging contaminants (ECs) are described as chemical compounds that have adverse effects on the health of humans, animals, and the environment. Despite their negative impact, these compounds are still essentially unregulated, and their fate in the environment is still not sufficiently understood (O’ Flynn et al., 2021). ECs are inclusive of pharmaceuticals, hormone-regulating medications, and their relative metabolites. The effects of human influence in combination with these ECs have detrimental consequences on surface water as stated in previous research (Archer, 2018; Aris et al., 2014).

Pharmaceuticals are synthesised to be stable by nature and exhibit their required biological effects at relatively low concentrations—characteristics of which consequently lead to environmental persistence. A major source of pharmaceutical contamination in surface water is from effluent discharged from wastewater treatment plants (WWTPs). The rate at which these pharmaceuticals enter the surface water is much higher than the rate of degradation, and this leads to a pseudo-persistence (Archer, 2018). This phenomenon is the result of rapid urbanisation and exponential population growth which has caused wastewater treatment plants to operate beyond their original design capacity. Globally, municipal WWTPs primarily deal with wastewater arising from domestic, medical, and industrial sources; however, it is common for stormwater runoff to also be treated at WWTPs.

The most common type of WWTPs situated in urban areas make use of the activated sludge treatment method for the removal of various compounds. This is inclusive, but not limited to, nutrients and inorganics. This method is efficient as it ensures high removal of nutrients, organic matter, as well as suspended solids at relatively low operational costs (O’ Flynn et al., 2021). However, this treatment process is not specifically tailored to remove pharmaceuticals and other ECs from wastewater, resulting in their presence in the treated effluent from WWTPs. Removal efficiencies observed at various WWTPs depend on numerous factors, some of which include the physiochemical properties of the compound, climate conditions (such as temperature, sunlight intensity, and rainfall), and operational conditions of the treatment process (relating to temperature, redox conditions, and retention times) in addition to the age and condition of the activated sludge (Gracia-Lor et al., 2012). Prior studies undertaken by Mines et al. (2007) showed strong correlations between rainfall intensity and WWTP flow rates, thus indicating the extent to which meteorological conditions influence the treatment processes conducted at WWTPs (Mines et al., 2007). As a result of these factors, removal efficiencies are observed to vary significantly among WWTPs, as well as within a specific plant at different time intervals (Gracia-Lor et al., 2012). It is expected that during the dry seasons, high concentrations of pollutants entering the WWTP are sufficiently removed by the WWTP process, while influents that are diluted with rain/stormwater experienced during the rainy, wet seasons are found to cause operational shortcomings and thus lower treatment efficiency (McMahan, 2006); however, it is important to note that the impact of rainfall would affect each WWTP differently. Heavier rainfall may prove to be beneficial to some plants and may negatively affect others. The type and capacity of individual unit processes within each WWTP, as well as various external factors, will be the major determining factors (Pocock and Joubert, 2018).

Although the vast majority of research done globally shows various ECs to be present in wastewater and receiving water bodies, the fate and impact that they have within treatment plants and environmental water bodies are still inadequately described. This study aims to identify and quantify analyte concentrations within various stages of the wastewater treatment process and subsequently determine relative removal efficiencies over two main seasons experienced within South Africa—the wet season (December–February) and the dry season (June–July). Additionally, the effects of a flooding event experienced during April 2022 on the efficiency of the wastewater treatment plant were investigated. Analytes selected for this study included pharmaceuticals used to treat various medical conditions that are prevalent in South Africa. Justification and relevant statistics to support the choice of pharmaceuticals are detailed in Inarmal (2022). These pharmaceuticals and their chemical properties are outlined in the Supplemental Information (SI – Table SI1). An additional aim of this study was to undertake an environmental risk assessment on the selected analytes and to determine whether they are of environmental concern. This is the first study showing removal efficiencies and risk quotients (RQs) of these analytes of interest at this particular WWTP. This study was conducted during the COVID-19 pandemic waves in South Africa, and the results provide information on analytes that were used more frequently during the pandemic, such as ivermectin, which has limited studies. Furthermore, it highlights the need for more risk analysis to be conducted especially on the analytes having a significantly high risk to the environment.

## Materials and methods

### Chemicals and reagents

Analytical standards purchased from Sigma-Aldrich, South Africa, and DLD Scientific included metformin, caffeine, sulfamethoxazole hydroxylamine, sulfamethoxazole, primidone (internal standard), nevirapine, prednisolone, valsartan, rifampicin, 17α-ethynylestradiol (EE2), and ivermectin. Millipore water was acquired from a Synergy ® UV water purification system. HPLC grade methanol, ammonium acetate, and acetic acid were purchased from Sigma-Aldrich, South Africa. Oasis® HLB cartridges (60 mg, 3 cc) were purchased from Microsep (Pty) Ltd.

### Study site and sample collection

**Fig. 1:**
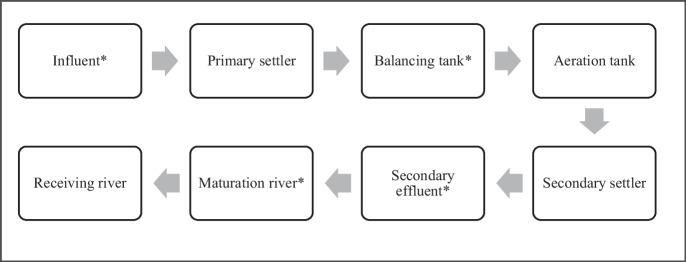
Simple schematic showing basic stages of wastewater treatment. *Sampling sites

The study site for this research endeavour was a Wastewater Treatment Plant, situated in Pietermaritzburg, KwaZulu-Natal, South Africa. The WWTP had a mean influent flow rate of 100 ML day^−1^ and, in 2022, serviced an immediate Pietermaritzburg population of approximately 539,069 people (World Population Review, 2024). Flow rate refers to the rate at which raw wastewater enters the treatment plant as well as the rate treated wastewater exits the plant on a daily basis; this is measured as mega litres (ML) entering or exiting the plant in a given day and is expressed as ML day^−1^. Wastewater samples were collected once daily from four sampling sites within the plant, over a period of seven consecutive days. Samples were taken over three sampling campaigns: February 2022, April 2022, and June 2022, with each campaign representing the wet season, flooding event, and dry season, respectively. Sampling points included the inlet, balancing tank, secondary effluent, and maturation river. A simple schematic diagram illustrating the basic stages of wastewater treatment followed by the WWTP is shown in Fig. 1. Upon collection, wastewater samples were acidified with three drops of acetic acid until a pH of 7 was reached. The samples were kept on ice during transportation to the laboratory. Once at the laboratory, samples were stored in the fridge at 4 °C. Filtration and extraction of all samples were done within 2 weeks of sample collection.

### Method development and method validation

#### Recovery studies

Recovery studies formed part of an investigation to determine whether the experimental procedure applied to the water samples was efficient in the extraction of targeted analytes. This was carried out by spiking and extracting analytes from a tap water sample using the method described in the “Solid phase extraction (SPE) of analytes of interest” section. An additional tap water sample, not spiked with any standard, was extracted through the same methods described in the “Solid phase extraction (SPE) of analytes of interest” section. During this investigation, various parameters were modified to ensure the maximum percentage recovery of the target analytes was achieved and ensured that analytes did not adsorb to the Whatman filter paper. Percentage recovery was calculated using Eq. 1. Acceptable recoveries fell between the range of 73.53 to 105.80%. The results obtained are outlined in Table 1.

**Table 1:** Method validation data obtained for analytes of interest

Pharmaceutical	LOD (mg L^−1^)	LOQ (mg L^−1^)	Recovery (%)	Interday (%RSD)	Intraday (%RSD)	*R*^2^
Metformin	0.322	0.974	83.17	3.75	2.63	0.9909
Caffeine	0.033	0.099	86.42	2.32	1.47	0.9936
Sulfamethoxazole hydroxylamine	0.071	0.214	79.65	4.52	3.22	0.9957
Sulfamethoxazole	0.072	0.219	73.53	0.96	1.58	0.9942
Nevirapine	0.066	0.200	100.70	1.36	1.61	0.9971
Prednisolone	0.886	2.684	95.56	1.05	1.89	0.9970
Valsartan	0.220	0.667	89.61	1.84	1.91	0.9950
Rifampicin	0.062	0.187	82.33	0.56	1.31	0.9933
EE2	0.215	0.653	105.80	1.21	1.07	0.9938
Ivermectin	0.589	1.784	88.35	1.54	1.63	0.9952

1 $${\% recovery}= \frac{\text{spiked sample concentration}-\text{unspiked sample concentration}}{\text{concentration used to spike}}\times 100$$

#### Limit of detection (LOD) and limit of quantification (LOQ)

LOD refers to the lowest concentration of an analyte that can be detected via an analytical method, while LOQ refers to the lowest concentration at which the performance of an analytical method is acceptable for usage. Equations 2 and 3 were used to calculate these parameters:

2 $$\mathrm{LOD }=3.3\times \frac{\text{standard deviation}}{\mathrm{gradient}}$$

3 $$\mathrm{LOQ }=10\times \frac{\text{standard deviation}}{\mathrm{gradient}}$$

Analysis of standards was done in triplicate. Calibration curves were generated for each replicate, and the standard range was made in pure methanol. These curves were drawn using the peak area from the resulting chromatograms in relation to the standard concentrations. The standard deviation that exists between the y-intercept values of each equation is used as well as the average gradient. Results obtained are depicted in Table 1.

#### Interday and intraday analyses

An interday study is undertaken by performing an analysis multiple times over a series of days, while intraday studies relate to undertaking an analysis multiple times within a single day. Interday and intraday analyses aim to determine the ability of an instrument to reproduce consistent data output and are expressed as percentage relative standard deviation (%RSD). To ensure reliable reproducibility, %RSD values for interday and intraday analyses should be below 10% (UNODC, 2009). The results outlined in Table 1 depict all results being below 10%. This is indicative of consistency in the instrument’s ability for the analysis.

### Solid phase extraction (SPE) of analytes of interest

Wastewater samples were initially gravity filtered using Whatman No. 40 filter paper to remove larger particles, such as scum. Thereafter, a volume of 50 mL was further filtered using a 0.45-µm filter disc, and the pH of the resulting sample was adjusted to pH 7 using acetic acid (Archer et al., 2017). An Oasis® HLB cartridge was used to conduct SPE. Cartridges were conditioned with 2 mL of methanol and followed by 2 mL of Millipore water. The double filtered water sample was then loaded onto the cartridge and was allowed to pass through at a flow rate of approximately 2 mL min^−1^. SPE cartridges were then dried under vacuum for a period of approximately 15 min. Extracts were then eluted with 4 mL of methanol into amber glass vials. The eluate was evaporated to dryness under vacuum and thereafter reconstituted in 1 mL using a 6 mg L^−1^ standard solution (comprising *all* analytes of interest in methanol), which was used to spike the samples. This method of spiking was used to bring the concentration of the analytes being measured to a level greater than the LOQ of the instrument. This was performed by adding a precise volume (1 mL) of a known analyte solution (6 mg L^−1^) to the sample. This concentration was then subtracted from the concentration determined to give the final true concentration of the analyte in the sample. To further ensure no carryover of analyte between sample analyses, a blank methanol sample was run between samples. In addition, a 6 mg L^−1^ standard mixture was analysed between intervals to ensure sample analysis was consistent.

### Liquid chromatography-mass spectrometry (LC–MS) analysis of analytes of interest

A Shimadzu LC–MS-2020 system was used for the analysis of targeted analytes following a method adapted from Archer (2018). A Shimadzu Shim-pack GIST reversed phase C18 column was used, with 2.1 × 100 mm dimensions and 3-µm particle size. Mobile phases used for the analysis were (A) Millipore water consisting of 5 mM ammonium acetate and 3 mM acetic acid and (B) 100% high pressure liquid chromatography (HPLC) grade methanol. The mobile phase flow rates and injection volumes were kept consistent at 0.22 mL min^−1^ and 5 µL, respectively. Method blanks containing methanol were inserted after each sample run. Gradient elution was performed by the following method: 0–1 min, 20% B isocratic hold; 1–18.50 min, from 20% B to 90% B; 18.50–22.50 min, 90% B isocratic hold; 22.50–25 min, 90% B–20% B; and 25–27.50 min, 20% isocratic hold to recondition the column to initial conditions. The mass spectrometer had a single quadrupole mass analyser and electrospray ionisation (ESI). All analytes were ionised under ESI positive mode. The ions were detected in scan mode with a *m/z* scan range of 100–900 m*/z*. Only the molecular ion peaks for all analytes were monitored. These are outlined in Table 2. The analyte molecular ion, together with the known retention times of each analyte from analysis of the standard mixture, was used to confirm the identification of the analytes of interest. The ionisation chamber and detector voltages were − 3.50 kV and − 1.45 kV, respectively, with an ion source temperature of 200 °C.

**Table 2:** Molecular ion *m/z* for analytes of interest

Analyte	Molecular ion (*m/z*)
Metformin	130
Caffeine	195
Sulfamethoxazole hydroxylamine	270
Sulfamethoxazole	254
Nevirapine	267
Prednisolone	361
Valsartan	436
Rifampicin	823
EE2	296
Ivermectin	877

## Results and discussion

### Concentrations of analytes within the various stages of the treatment process

**Fig. 2:**
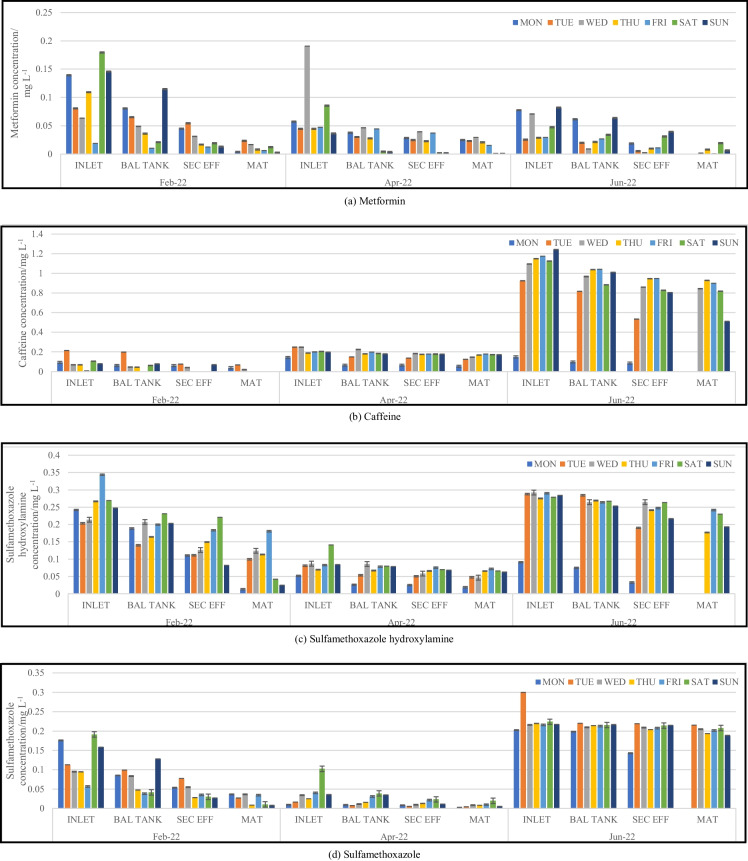

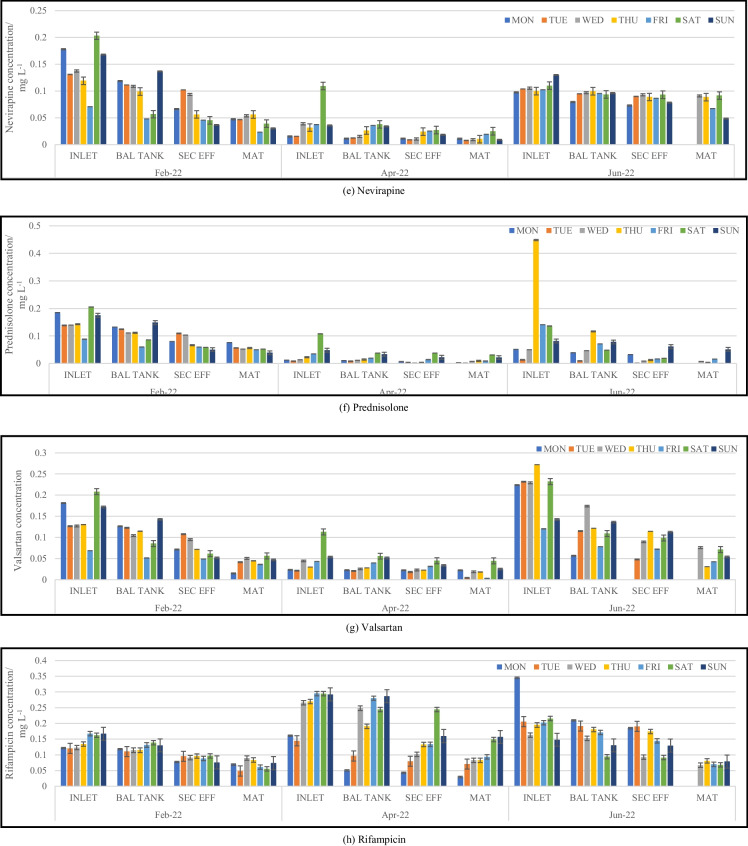

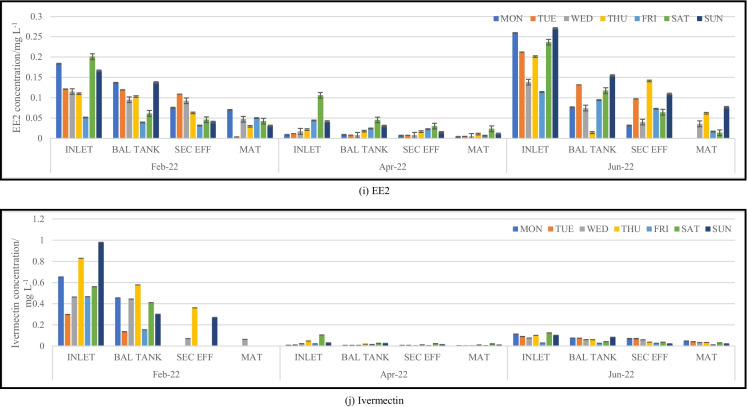
Trends observed for pharmaceutical concentrations within the plant process during sampling trips. BAL TANK, balancing tank; SEC EFF, secondary effluent; MAT, maturation river

Wastewater samples were collected once a day over a period of seven consecutive days. Figure 2a–j illustrates analyte concentrations at stages of the treatment process that were sampled. Concentrations are expressed in mg L^−1^.

There was an observable change in the concentration of the analytes between samples collected from the influent and effluent of the wastewater treatment plant. The highest concentrations of analytes were detected in the inlet samples, with only a slight decrease in the analyte concentrations of the wastewater samples collected from the balancing tank. The wastewater sampled from the secondary effluent showed a minimal reduction in concentration compared to that found in the balancing tank despite the wastewater being treated with activated sludge. This could be due to the sludge mainly being able to target the digestion of nutrients and the breakdown of organics such as proteins, fats, and carbohydrates, rather than complex chemical compounds such as pharmaceuticals (Navalon et al., 2011). In most cases, a significant reduction in pharmaceutical concentration can be observed in the maturation river samples. This could be attributed to the treated wastewater being exposed to the elements of nature during its flow from the maturation river into the river to which it is discharged. The maturation river flows for approximately 7 km before discharge into the Msunduzi River. As the open maturation river flows, it is exposed to ultraviolet (UV) rays from the sun. This phenomenon could ultimately lead to photodegradation of many pharmaceuticals which is confirmed from the significantly lower concentrations found from this sampling site. A study undertaken by Kawabata et al. (2013) investigated the effects that sunlight has on a range of pharmaceuticals found within aquatic media. The results concluded that sunlight (predominantly UV-B radiation) was responsible for the partial degradation of several pharmaceutical compounds. The complete photodegradation of a few pharmaceuticals did occur; however, the variation is majorly due to the chemical structure of the compounds and the presence of certain functional groups (Kawabata et al., 2013). At the WWTP, the maturation river flows for a few kilometres within a man-made cement or equivalent channel. This therefore creates an increased surface area for the photodegradation of pharmaceuticals. Adsorption or leaching into the soil and sediment surrounding the channel is a possibility; however, if adsorption or leaching into sediment were to occur along the maturation river channel, it could happen at any point within the treatment process and not exclusively at this point as all the channels and pipes within the plant are made from similar materials. Further studies analysing soil and sediment from within the WWTP are an area for investigation.

Analyte concentrations quantified within the maturation river were significantly lower than that from the inlet; however, the mere presence of certain compounds such as antibiotics and antituberculosis pharmaceuticals allows for the development of drug-resistant bacteria. Concentrations of sulfamethoxazole hydroxylamine (metabolite) and sulfamethoxazole (parent) detected in the maturation river samples ranged from not detected to 0.2298 mg L^−1^ and not detected to 0.2152 mg L^−1^, respectively. While there are no published South African guidelines for these analytes in wastewater, prior studies have shown that antibiotics should ideally be present in concentrations below 1.0000 × 10^−5^ mg L^−1^ to avoid the development of resistant bacterial strains (Le Page et al., 2017). Caffeine concentrations detected in the inlet samples ranged from 0.007587 to 1.243 mg L^−1^ and between not detected and 0.9286 mg L^−1^ in the maturation river. Caffeine at high concentrations has been found to have the potential to alter physiochemical features within soil and water organisms (Al-Janabi, 2011). Selected bacterial species use caffeine as a significant source of carbon to meet their nutritional requirements; however, other species experience repressed growth rates and inhibited metabolic processes when in the presence of caffeine (Al-Janabi, 2011).

Concentrations of EE2 quantified within the inlet samples ranged from 0.008684 to 0.2701 mg L^−1^ compared to the maturation river sample concentrations of not detected to 0.07638 mg L^−1^. Despite a significant decline in the concentration levels, the presence of EE2 in aquatic environments could potentially lead to the feminization of male aquatic organisms and infertility among the female population due to its ability to bioaccumulate (Aris et al., 2014). This is also possible at concentration levels as low as ng L^−1^ and would ultimately lead to the extinction of various aquatic species over time as a result of the inability to reproduce. Concentrations of metformin, nevirapine, prednisolone, and valsartan within the inlet samples ranged from 0.02550 to 0.1905 mg L^−1^, 0.01522 to 0.2031 mg L^−1^, 0.008775 to 0.4482 mg L^−1^, and 0.02129 to 0.2719 mg L^−1^, respectively. While maturation river concentrations ranged from not detected to 0.02939 mg L^−1^ for metformin, not detected to 0.09092 mg L^−1^ for nevirapine, not detected to 0.07650 mg L^−1^ for prednisolone, and not detected to 0.07589 mg L^−1^ for valsartan. The concentration of ivermectin quantified in the inlet samples ranged from 0.008184 to 0.9793 mg L^−1^ and for the maturation river samples from not detected to 0.06237 mg L^−1^. Prior studies have shown that the bioaccumulation of ivermectin in the tissue of aquatic organisms can reduce motor activity and affect the rate of reproduction and growth (Ding et al., 2001).

### Mass loads

Wastewater influent and effluent flow rates differ among treatment plants as well as within a single treatment plant at different times of the day. The flow rate is dependent on various factors such as rainfall, variation in the population serviced by the WWTP, and water usage by the population within the area. Mass loads are therefore calculated to compensate for the varying flow rates at the treatment plant influent and effluent streams. It is calculated using Eq. 4 and represented as g day^−1^. Calculated mass loads are outlined in the SI (Table SI 2).

4 $$\text{Mass loads}=\text{calculated influent or effluent concentration }\left(\frac{\mathrm{mg}}{\mathrm{L}}\right)\times \text{flow rate}\left(\frac{\mathrm{ML}}{\mathrm{day}}\right)\times 1000$$

The conversion factor of 1000 is derived from Eq. 5. The use of conversions is essential to convert concentration units $\left(\frac{\mathrm{mg}}{\mathrm{L}}\right)$ and flow rate units $\left(\frac{\mathrm{ML}}{\mathrm{day}}\right)$ into the desired units for the measurement of mass loads $\left(\frac{\mathrm{g}}{\mathrm{day}}\right)$.

5 $$\frac{\mathrm{mg}}{\mathrm{L}}\times \frac{\mathrm{ML}}{\mathrm{day}}\times \frac{1\mathrm{ g}}{\text{1,000 mg}}\times \frac{\text{1,000,000 L}}{1\mathrm{ ML}}$$

The flow rate refers to the rate at which raw wastewater enters the treatment plant as well as the rate at which treated wastewater exits the plant on a daily basis; this is expressed as ML day^−1^. Table 3 outlines the average daily influent and effluent flow rates recorded at the WWTP. Additionally, the daily average rainfall experienced in the area serviced by the WWTP, Pietermaritzburg, is also shown.

**Table 3:** Average daily influent and effluent flow rates and rainfall data for sampling period

Date	Inflow (ML day^−1^)^a^	Outflow (ML day^−1^)^a^	Average rainfall^b^ (mm)
31/01/2022	90	86	0.0
1/02/2022	75	71	0.8
2/02/2022	86	82	2.8
3/02/2022	76	72	1.7
4/02/2022	83	79	15.4
5/02/2022	93	89	5.8
6/02/2022	103	99	4.9
18/04/2022	112	107	0.6
19/04/2022	112	107	0.0
20/04/2022	110	105	0.0
21/04/2022	105	101	0.0
22/04/2022	114	109	0.0
23/04/2022	90	85	0.2
24/04/2022	116	110	1.1
6/06/2022	66	63	0.1
7/06/2022	70	67	0.0
8/06/2022	67	64	0.0
9/06/2022	67	64	0.0
10/06/2022	76	72	0.0
11/06/2022	71	68	0.0
12/06/2022	67	64	0.0

^a^Average daily inflow and outflow flow rates were provided by the Wastewater Treatment Plant

^b^World Weather Online (2022)

From the results depicted in the SI (Table SI 2), it was observed that mass loads of each pharmaceutical fluctuated with the seasonal use of that pharmaceutical. For example, mass loads of caffeine were found to be the lowest during the February 2022 sampling campaign and highest during the June 2022 sampling campaign. South Africa experiences its summer during the months of December–February each year. The hot weather experienced during this time period implies that high caffeine-containing beverages such as coffee would not be consumed during this time. Instead, caffeine-containing beverages such as coffee and tea would be consumed more regularly during the cold, winter months which are experienced between June and July each year. Other trends observed showing the seasonal use of each pharmaceutical are included in a previous study undertaken by Inarmal and Moodley (2023).

According to Table 3, rainfall potentially influences the daily flow rate of wastewater into the WWTP. During June 2022 (dry season), the lowest overall flow rates were observed with an average weekly rainfall of 0.1 mm recorded. The highest flow rates were recorded for April 2022 (flooding event), ranging from 85 to 116 ML day^−1^. However, a total of 1.9 mm of rainfall was recorded for this week. This could have resulted from the April 2022 flood experienced in KwaZulu-Natal that occurred a week before samples were collected. An estimated average of around 200 mm of rainfall was experienced in the province during this time, and as a result, WWTPs would have experienced significantly higher inflow, mainly comprising of stormwater and surface water runoff. The sampling period during February 2022 (wet season) was recorded to have had intermediate flow rates, when compared to that of April and June.

### Removal efficiencies

Removal efficiency, Eq. 6, indicates the amount of analyte that is removed during the treatment process and is expressed as a percentage. The abbreviations ML (influent) and ML (effluent) refer to the calculated mass load for an analyte in the influent and effluent stream, respectively. The equation is multiplied by 100 to express the result as a percentage.

6 $$\text{Removal efficiency}=\frac{\mathrm{ML }\left(\mathrm{influent}\right) -\mathrm{ ML }\left(\mathrm{effluent}\right) }{\mathrm{ML }\left(\mathrm{influent}\right)} \times 100$$

Removal efficiencies calculated for each sampling period are outlined in Table 4. The weekly averages are also shown for each analyte to provide an overall representation of the removal efficiency of the treatment process.

**Table 4:** Removal efficiencies obtained for sampling periods

Date	Removal efficiency (%)									
	MET	CAF	SMX N–OH	SMX	NEV	PRED	VAL	RIF	EE2	IVE
31/01/2022	97.28	60.54	94.82	80.26	74.66	60.56	92.07	45.78	63.50	> 99.99
1/02/2022	72.57	70.32	53.67	77.44	66.31	61.16	68.94	61.69	97.03	> 99.99
2/02/2022	74.64	72.26	44.57	63.15	62.64	64.30	62.08	30.42	60.92	87.15
3/02/2022	92.97	> 99.99	59.62	91.43	55.28	62.63	67.38	41.05	74.55	> 99.99
4/02/2022	67.15	> 99.99	49.95	41.80	68.41	45.91	49.79	65.17	7.70	> 99.99
5/02/2022	93.30	> 99.99	84.95	94.84	81.67	75.56	74.02	67.32	79.77	> 99.99
6/02/2022	98.15	> 99.99	90.33	95.18	82.71	78.53	73.77	57.61	82.31	> 99.99
Weekly average	85.15	85.59	68.27	77.73	70.24	64.09	69.72	52.72	66.54	97.31
18/04/2022	58.72	63.96	65.56	80.83	30.96	77.13	8.08	82.39	61.65	55.04
19/04/2022	50.25	51.52	43.89	73.98	51.79	94.01	80.65	53.31	59.64	82.90
20/04/2022	85.27	43.49	48.77	76.52	77.35	43.33	59.84	70.19	70.70	89.68
21/04/2022	54.67	14.59	9.10	69.04	69.05	61.09	41.15	70.45	52.63	78.84
22/04/2022	69.12	14.69	17.14	76.63	51.49	75.01	92.62	69.59	85.91	82.83
23/04/2022	99.72	20.63	55.84	81.68	78.53	72.87	62.72	52.16	79.04	81.58
24/04/2022	98.35	16.95	29.13	86.09	76.35	57.32	55.45	49.01	74.80	68.30
Weekly average	73.72	32.26	38.49	77.83	62.22	68.68	57.22	63.87	69.20	77.03
6/06/2022	> 99.99	> 99.99	> 99.99	> 99.99	> 99.99	> 99.99	> 99.99	> 99.99	> 99.99	59.85
7/06/2022	> 99.99	> 99.99	> 99.99	31.34	> 99.99	> 99.99	> 99.99	> 99.99	> 99.99	57.51
8/06/2022	97.75	26.30	> 99.99	9.35	17.45	85.61	68.36	60.57	75.31	58.25
9/06/2022	73.31	22.81	38.64	15.87	15.30	99.43	89.14	60.44	70.81	66.19
10/06/2022	99.07	27.41	21.11	11.62	37.58	89.26	66.39	67.20	86.00	59.77
11/06/2022	60.25	30.35	21.04	11.17	20.28	99.53	70.52	69.63	94.35	76.30
12/06/2022	92.35	60.77	34.92	17.14	64.35	40.20	63.81	49.21	72.99	80.02
Weekly average	88.67	52.23	58.96	27.93	50.42	87.43	79.46	72.15	85.35	65.41

*MET* metformin, *CAF* caffeine, *SMX N–OH* sulfamethoxazole hydroxylamine, *SMX* sulfamethoxazole, *NEV* nevirapine, *PRED* prednisolone, *VAL* valsartan, *RIF* rifampicin, *EE2* 17α-ethynylestradiol, *IVE* ivermectin

From Table 4, February, despite being the wet season, was determined to have the highest average removal efficiency. During this time, intermediate flow rates were recorded, as shown in Table 3, ranging from 75 to 103 ML day^−1^, and the percentage of analytes removed during the treatment process ranged from 7.70 to > 99.99%. A percentage of > 99.99% removal relates to the analyte not being detected during LC–MS analysis of the maturation river samples as this implies that the concentration of the analyte in the sample was below the limit of detection (LOD) of the instrument, and it would not be statistically correct to assume that 100% of the analyte was removed. Prior studies have indicated that non-detection of an analyte does not necessarily imply that complete removal has occurred and hence the alleviation of associated risks but could simply suggest conversion of the analyte into transformation products within the sample has occurred (Yadav et al., 2017). The lowest average removal efficiency was determined from the April samples, and this could be attributed to the higher flow rates observed in the treatment plant. During the month of April 2022, the province of KwaZulu-Natal experienced heavy than usual rainfall that resulted in flooding of various regions. The heavier than usual inflow into the WWTP would have resulted in ineffective removal of pollutants due to the pollutants not spending sufficient time in the wastewater process during the treatment process.

Removal efficiencies observed for June, which is described as the dry season, ranged from 9.35 to > 99.99% with the lowest daily average flow rate recorded, ranging from 63 to 76 ML day^−1^. This could be an indication that slower flow rates allow for more contact time with each stage of the treatment process and possibly result in higher analyte removal. However, there are various additional factors that could affect the removal of analytes from a wastewater stream, such as the age, pH, and temperature of the activated sludge used for the treatment process. Biomass concentrations, other meteorological conditions, and interruption within the plant due to maintenance or loadshedding also affect the removal efficiency. The pharmaceutical with the highest average removal efficiency was ivermectin (97.31%), established for the February 2022 sampling period, and the lowest average was sulfamethoxazole (27.93%), quantified for the June 2022 sampling period. It is not possible for wastewater treatment processes to remove all types of pharmaceutical or chemical compounds in use today as there is a substantial variety available with most having vastly different chemical structures and properties. Concentration reductions observed between the influent and effluent streams could also be attributed to biodegradation and sludge sorption during wastewater treatment or adsorption onto sediment within the plant (Park et al., 2020). Sludge treatment of wastewater normally occurs between the balancing tank and the secondary effluent sampling positions. The results of the study suggest that maximum removal of analytes was achieved between the secondary effluent and the maturation river and not post-sludge treatment. A study undertaken by Archer (2018) revealed removal efficiencies for sulfamethoxazole (18%), valsartan (90%), metformin (93%), and caffeine (100%) from a WWTP in Western Cape, South Africa. With the exception of sulfamethoxazole, the Western Cape WWTP had an overall higher removal efficiency, averaging at 80% removal, for most pharmaceuticals and metabolites (Archer, 2018). This could be attributed to the Western Cape WWTP being in a better condition, with optimally functioning infrastructure, compared to that in this study.

Significant changes in removals were also attributed to the flooding that occurred within the province during the time of the study. The heavy rainfall would have resulted in the dilution of analyte concentrations. This would have ultimately resulted in a lower concentration being removed by the wastewater treatment process during the flooding period. Furthermore, results from this study suggest that the plant was not functioning optimally, and this is noticeable in the low removal efficiencies observed. Optimum functioning would have been significantly reduced due to external factors such as extended periods of loadshedding experienced at the water treatment plant in April 2022 due to infrastructure damage.

### Environmental risk assessment

Environmental risk assessment (ERA) is a scientific process used to identify and evaluate the likelihood of a chemical compound to threaten living organisms, natural habitats, and ecosystems. It is determined by considering the predicted no-effect concentration (PNEC), which is taken from literature, and the measured environmental concentration, as shown in the effluent stream of this study (MEC). The effluent concentration was taken as the MEC as this is the wastewater that is being discharged into the Msunduzi River. PNEC refers to the maximum concentration of a compound that will exhibit no adverse effects on an organism or ecosystem. ERA, Eq. 7, is calculated by determining the ratio of the MEC value to PNEC. A risk quotient (RQ) value greater than or equal to 1 implies that the chemical compound is of environmental concern. Table 5 outlines the PNEC values obtained from various literature sources and the calculated ERA ratio for each analyte.

**Table 5:** ERA ratios obtained for each pharmaceutical

Pharmaceutical	PNEC (mg L^−1^)	Reference	ERA ratio		
			February 2022	April 2022	June 2022
MET	0.060	Bergman et al. (2011)	0.177	0.273	0.0863
CAF	0.011	Bergman et al. (2011)	1.63	13.2	51.9
SMX N–OH	ND	ND	ND	ND	ND
SMX	5.9 ${\times 10}^{-4}$	Bergman et al. (2011)	39.1	14.0	293
NEV	8.0 ${\times 10}^{-5}$	Cid et al. (2021)	529	162	690
PRED	2.3 ${\times 10}^{-4}$	Ren et al. (2022)	238	51.3	48.4
VAL	0.090	Van der Aa et al. (2011)	0.463	0.217	0.436
RIF	9.1 ${\times 10}^{-5}$	Fass (online)	756	10.4 ${\times 10}^{2}$	572
EE2	1.0 ${\times 10}^{-8}$	Bergman et al. (2011)	3.90 ${\times 10}^{6}$	9.30 ${\times 10}^{5}$	2.91 ${\times 10}^{6}$
IVE	3.0 ${\times 10}^{-11}$	Bergman et al. (2011)	29.7 ${\times 10}^{7}$	25.4 ${\times 10}^{7}$	10.5 ${\times 10}^{8}$

*ND* no data available, *MET* metformin, *CAF* caffeine, *SMX N–OH* sulfamethoxazole hydroxylamine, *SMX* sulfamethoxazole, *NEV* nevirapine, *PRED* prednisolone, *VAL* valsartan, *RIF* rifampicin, *EE2* 17α-ethynylestradiol, *IVE* ivermectin

7 $$\text{Risk quotient}=\frac{\mathrm{MEC}}{\mathrm{PNEC}}$$

The results in Table 5 were obtained by using the average weekly maturation river concentrations for each analyte. The maturation river is the final stage of the treatment process and flows directly into a nearby river. ERA ratios ranged from 0.0863 to 10.5 ${\times 10}^{8}$. A RQ ratio below 1 indicates that the analyte is not of environmental concern. Ratios obtained for metformin and valsartan were below 1 for each of the three sampling occasions. This corresponds with previous studies undertaken by Caldwell et al. (2019) and Archer (2018) respectively, whereby RQs for metformin and valsartan were found to be below 1.

The ERA ratios that are of moderate concern are those for caffeine, sulfamethoxazole, nevirapine, prednisolone, and rifampicin. Furthermore, according to Table 5, pharmaceuticals that are of significant concern are EE2 and ivermectin, as these compounds could have detrimental effects on aquatic organisms as they are not adequately removed by the wastewater treatment process. EE2 is classified as an endocrine disrupting compound (EDC) and hence has the potential for detrimental impact on aquatic life within the receiving river. Some of these effects would include the feminization of male species and infertility among female species which could potentially lead to the extinction of certain species over time (Aris et al., 2014). Prior studies done by Laurenson et al. (2014) on wastewater effluent from various WWTPs in the USA found maximum EE2 RQ values of 4.6. The RQs established for this study were significantly and alarmingly higher than those found by Laurenson et al. (2014). RQs observed from this study were significantly higher than worst-case RQs determined from a study undertaken by Liebig et al. (2010).

Although ERA studies are becoming more frequent, there is not enough information detailing the extent of the ecological and behavioural consequences that long-term exposure to these pharmaceuticals has on wildlife. Furthermore, it is important to note that pharmaceuticals can enhance the behaviour of other pharmaceuticals and organic compounds, often known as having a synergistic effect. This may also imply that many pharmaceuticals will enhance persistence within the receiving water, and this could ultimately lead to drug resistance in the case of antibiotic presence in the environment. As a result of the persistence of these pharmaceuticals, further in-depth studies are required due to their difficult removal and significant risk to the environment.

## Conclusion

This study aimed to identify and quantify analyte concentrations within various stages of the wastewater treatment process during the wet and dry seasons experienced in KwaZulu-Natal, as well as investigate the impact that a flooding event has on WWTP efficiency. Furthermore, the study aimed to determine the removal efficiencies of those analytes. Influent concentrations of analytes ranged from 0.008184 to 1.243 mg L^−1^ while maturation river concentrations ranged from not detected to 0.9286 mg L^−1^. Removal efficiencies ranged from 7.70 to > 99.99% of analyte removed. The investigation demonstrated that majority of analyte removal occurred between the secondary settler and the maturation river. There were noticeable seasonal trends observed; however, the occurrence of the April 2022 flood in the province had significantly impacted WWTP infrastructure, and this is observed by the significantly lower removal efficiencies during the April 2022 and June 2022 sampling campaigns.

In addition, an environmental risk assessment on the selected analytes and determination of whether they are of environmental concern was performed. The research study has highlighted the trend of increasing pharmaceutical usage and the relative increase in mass loads found in wastewater. The pharmaceuticals selected for this study showed varying removal patterns during the wastewater treatment process, some of which being almost completely removed and others showing effluent concentrations comparable with influent concentrations. Environmental risk assessment ratios ranged from 0.0863 to 10.5 ${\times 10}^{8}$. Ratios for metformin and valsartan were well below 1, indicating that they would not be of environmental risk. While ratios for ivermectin were substantially high, ranging from 25.4 ${\times 10}^{7}$ to 10.5 ${\times 10}^{8}$ indicating that it is of severe environmental risk and is of significant concern. EE2 also showed increased environmental risk and highlights the need for further investigation into its presence in the environment.

## Supplementary Information

Below is the link to the electronic supplementary material.

Supplementary file1 (DOCX 34 KB)

## Data Availability

The authors declare that the data supporting the findings of this study are available within the paper and its Supplementary Information files.
