# Supplementary material for: Removal efficiencies and environmental risk assessment of selected pharmaceuticals and metabolites at a wastewater treatment plant in Pietermaritzburg, South Africa
Source: Environ Monit Assess. 2024 Dec 26;197(1):102. doi: 10.1007/s10661-024-13515-z (PMC11671546; doi:10.1007/s10661-024-13515-z)
Supplement: Supplementary file 1 — Supplementary file1 (DOCX 34 KB) [file 10661_2024_13515_MOESM1_ESM.docx]

**Supplemental Information**

**REMOVAL EFFICIENCIES AND ENVIRONMENTAL RISK ASSESSSMENT OF SELECTED PHARMACEUTICALS AND METABOLITES AT A WASTEWATER TREATMENT PLANT IN PIETERMARITZBURG, SOUTH AFRICA**

Nikitha Inarmal and Brenda Moodley*

School of Chemistry and Physics, University of KwaZulu-Natal, Westville Campus, 4000, Durban, South Africa

E-mail first author: [Nikki.inarmal@icloud.com](mailto:Nikki.inarmal@icloud.com)

ORCID: 0000-0002-8205-1607

*E-mail corresponding author: [moodleyb3@ukzn.ac.za](mailto:moodleyb3@ukzn.ac.za)

ORCID: 0000-0002-0146-1138

**Table SI 1:** Chemical properties of pharmaceuticals selected for this study

**Table SI 2:** Calculated mass loads.

**Table SI 1**: Chemical properties of pharmaceuticals selected for this study

| **Class** | **Compound** | **Molecular formula^a^** | **Molar mass^a^/**  **g mol^-1^** | **Melting point^a^/⁰C** | **Boiling point^b^/⁰C** | **Solubility^a^/mg mL^-1^** |
| --- | --- | --- | --- | --- | --- | --- |
| Diabetes | Metformin | C_4_H_11_N_5_ | 129.16 | 224.5 | 172.5 | 1.38 |
| Stimulant | Caffeine | C_8_H_10_N_4_O_2_ | 194.19 | 236.5 | 178.0 | 16 |
| Antibiotic | Sulfamethoxazole hydroxylamine | C_10_H_11_N_3_O_4_S | 269.28 | 130.0 | 502.2 | - |
|  | Sulfamethoxazole | C_10_H_11_N_3_O_3_S | 253.28 | 169.0 | 482.1 | 0.50 |
| Antiretroviral | Nevirapine | C_15_H_14_N_4_O | 266.30 | 248.0 | 415.4 | 0.00071 |
| Endocrine disruptors | Prednisolone | C_21_H_28_O_5_ | 360.44 | 235.0 | 570.6 | 0.223 |
|  | 17α-ethinylestradiol | C_20_H_24_O_2_ | 296.40 | 143.0 | 457.2 | 0.0113 |
| Hypertension | Valsartan | C_24_H_29_N_5_O_3_ | 435.52 | 116.5 | 85.5 | 0.00141 |
| Tuberculosis | Rifampicin | C_43_H_58_N_4_O_12_ | 822.94 | 185.5 | 937.0 | 1.30 |
| Anthelmintic | Ivermectin | C_47_H_72_O­14­ | 875.10 | 155.0 | 940.4 | 0.004 |

^a^[1], ^b^[2], -: No data available.

**Table SI 2:** Calculated mass loads.

| **Pharmaceutical** | **Day of week** | **Mass loads/g day^-1^** | | | | | |
| --- | --- | --- | --- | --- | --- | --- | --- |
|  |  | **February 2022** | | **April 2022** | | **June 2022** | |
|  |  | **INFLUENT** | **EFFLUENT** | **INFLUENT** | **EFFLUENT** | **INFLUENT** | **EFFLUENT** |
| Metformin | Mon | 12508.18 | 339.70 | 6414.03 | 2647.91 | 5114.12 | 0.00 |
|  | Tues | 6048.75 | 1659.11 | 4993.07 | 2484.12 | 1785.09 | 0.00 |
|  | Wed | 5461.00 | 1384.73 | 20958.53 | 3086.18 | 4744.72 | 106.81 |
|  | Thurs | 8307.77 | 583.64 | 4671.64 | 2117.81 | 1932.25 | 515.78 |
|  | Fri | 1562.98 | 513.50 | 5407.55 | 1670.08 | 2229.61 | 20.81 |
|  | Sat | 16674.90 | 1116.95 | 7697.49 | 21.31 | 3365.94 | 1337.98 |
|  | Sun | 14997.77 | 277.20 | 4235.08 | 69.89 | 5501.91 | 420.90 |
| Caffeine | Mon | 8414.70 | 3320.36 | 16077.60 | 5794.05 | 9823.00 | 0.00 |
|  | Tues | 16032.55 | 4757.94 | 27774.10 | 13464.12 | 64690.20 | 0.00 |
|  | Wed | 5974.45 | 1657.27 | 27240.36 | 15393.00 | 73344.45 | 54052.81 |
|  | Thurs | 5265.60 | 0.00 | 19866.00 | 16968.00 | 76994.27 | 59431.86 |
|  | Fri | 629.73 | 0.00 | 22778.07 | 19432.36 | 89264.04 | 64799.15 |
|  | Sat | 9909.82 | 0.00 | 18447.73 | 14641.25 | 79883.57 | 55641.02 |
|  | Sun | 8185.86 | 0.00 | 22882.85 | 19004.15 | 83294.06 | 32676.53 |
| Sulfamethoxazole hydroxylamine | Mon | 21785.32 | 1128.39 | 5840.80 | 2011.60 | 6029.68 | 0.00 |
|  | Tues | 15262.50 | 7070.93 | 9077.60 | 5093.20 | 20147.42 | 0.00 |
|  | Wed | 18391.90 | 10194.12 | 9575.50 | 4905.23 | 19571.49 | 0.00 |
|  | Thurs | 20272.68 | 8185.94 | 7324.374 | 6658.03 | 18457.98 | 11326.04 |
|  | Fri | 28532.94 | 14282.14 | 9444.90 | 7826.20 | 22071.19 | 17411.04 |
|  | Sat | 25047.34 | 3769.15 | 12681.02 | 5599.66 | 19785.89 | 15623.63 |
|  | Sun | 25504.75 | 2466.74 | 9770.65 | 6924.50 | 19031.57 | 12384.93 |
| Sulfamethoxazole | Mon | 15837.14 | 3126.10 | 1099.64 | 210.79 | 13351.58 | 0.00 |
|  | Tues | 8456.25 | 1907.99 | 1848.00 | 480.87 | 21000.00 | 14419.02 |
|  | Wed | 8145.20 | 3001.20 | 3778.50 | 887.25 | 14453.63 | 13102.26 |
|  | Thurs | 7193.40 | 616.68 | 2647.23 | 819.50 | 14716.14 | 12381.30 |
|  | Fri | 4698.99 | 2734.68 | 4605.60 | 1076.10 | 16392.94 | 14488.41 |
|  | Sat | 17781.60 | 916.70 | 9214.20 | 1687.63 | 15899.49 | 14123.76 |
|  | Sun | 16325.65 | 787.05 | 4139.78 | 575.74 | 14566.25 | 12069.14 |
| Nevirapine | Mon | 16021.44 | 4059.20 | 1704.88 | 1177.00 | 6427.30 | 0.00 |
|  | Tues | 9821.25 | 3309.04 | 1742.16 | 839.95 | 7271.16 | 0.00 |
|  | Wed | 11820.70 | 4415.70 | 4265.69 | 966.00 | 7049.66 | 5819.43 |
|  | Thurs | 9047.54 | 4046.32 | 3321.25 | 1027.85 | 6688.89 | 5665.18 |
|  | Fri | 5884.70 | 1858.93 | 4280.70 | 2076.45 | 7777.20 | 4854.66 |
|  | Sat | 18892.95 | 3463.47 | 9838.71 | 2112.84 | 7802.29 | 6219.92 |
|  | Sun | 17295.19 | 2989.80 | 4139.07 | 979.00 | 8699.25 | 3101.10 |
| Prednisolone | Mon | 16682.24 | 6579.00 | 1398.65 | 319.93 | 3405.35 | 0.00 |
|  | Tues | 10372.50 | 4028.96 | 982.74 | 58.89 | 939.88 | 0.00 |
|  | Wed | 12001.30 | 4284.50 | 1495.45 | 847.41 | 3358.54 | 483.28 |
|  | Thurs | 10856.37 | 4057.20 | 2394.00 | 931.61 | 30030.49 | 170.90 |
|  | Fri | 7328.90 | 3964.03 | 3964.44 | 990.59 | 10707.56 | 1149.51 |
|  | Sat | 19083.60 | 4663.32 | 9713.53 | 2635.07 | 9672.23 | 45.11 |
|  | Sun | 18030.24 | 3870.90 | 5567.55 | 2376.00 | 5466.32 | 3269.11 |
| Valsartan | Mon | 16319.85 | 1294.30 | 2604.12 | 2393.59 | 14775.33 | 0.00 |
|  | Tues | 9487.50 | 2946.64 | 2385.02 | 461.42 | 16234.78 | 0.00 |
|  | Wed | 10930.60 | 4145.10 | 4902.01 | 1968.54 | 15348.96 | 4857.09 |
|  | Thurs | 9909.38 | 3232.80 | 3149.54 | 1853.60 | 18218.17 | 1978.33 |
|  | Fri | 5693.80 | 2858.65 | 4901.11 | 361.70 | 9156.37 | 3077.61 |
|  | Sat | 19339.35 | 5024.05 | 10189.29 | 3798.38 | 16499.81 | 4864.29 |
|  | Sun | 17718.81 | 4648.05 | 6203.88 | 2764.05 | 9530.90 | 3449.22 |
| Rifampicin | Mon | 10956.70 | 5940.40 | 18014.12 | 3171.94 | 22767.69 | 0.00 |
|  | Tues | 9040.22 | 3463.66 | 16215.06 | 7571.22 | 14400.45 | 0.00 |
|  | Wed | 10590.28 | 7369.05 | 29218.61 | 8709.12 | 10898.26 | 4297.66 |
|  | Thurs | 10213.12 | 6020.64 | 28310.31 | 8364.64 | 13068.91 | 5170.61 |
|  | Fri | 13890.50 | 4837.45 | 33599.36 | 10216.41 | 15355.61 | 5037.30 |
|  | Sat | 15072.84 | 4925.78 | 26497.08 | 12675.89 | 15293.11 | 4644.57 |
|  | Sun | 17231.31 | 7303.67 | 33887.52 | 17280.68 | 9907.24 | 5032.12 |
| EE2 | Mon | 16516.38 | 6028.60 | 972.63 | 372.97 | 17094.27 | 0.00 |
|  | Tues | 9097.50 | 270.29 | 1302.31 | 525.65 | 14850.11 | 0.00 |
|  | Wed | 9905.05 | 3870.40 | 1880.11 | 550.96 | 9254.17 | 2284.88 |
|  | Thurs | 8345.42 | 2123.67 | 2257.44 | 1069.25 | 13465.50 | 3930.24 |
|  | Fri | 4248.95 | 3921.87 | 5004.76 | 705.10 | 8656.14 | 1211.88 |
|  | Sat | 18693.00 | 3782.50 | 9519.52 | 1994.82 | 16786.99 | 948.23 |
|  | Sun | 17127.84 | 3029.79 | 4779.06 | 1204.32 | 18097.52 | 4888.69 |
| Ivermectin | Mon | 58727.32 | 0.00 | 954.28 | 429.02 | 7453.86 | 2992.50 |
|  | Tues | 22345.72 | 0.00 | 916.58 | 156.73 | 6335.00 | 2691.59 |
|  | Wed | 39819.48 | 5115.09 | 2620.53 | 270.39 | 5092.00 | 2126.11 |
|  | Thurs | 63001.30 | 0.00 | 5035.77 | 1065.71 | 6719.19 | 2271.84 |
|  | Fri | 38741.61 | 0.00 | 2490.95 | 427.59 | 2245.04 | 903.11 |
|  | Sat | 52177.39 | 0.00 | 9389.18 | 1729.40 | 8846.24 | 2096.76 |
|  | Sun | 100876.20 | 0.00 | 3669.30 | 1163.12 | 6823.37 | 1363.20 |

**References:**

[1] PubChem. [Online]. Available: https://pubchem.ncbi.nlm.nih.gov/. [Accessed 16 August 2022].

[2] ChemSpider. [Online]. Available: http://www.chemspider.com/. [Accessed: 22nd August 2022].
